# Giving the Giggles: Prediction, Intervention, and Young Children's Representation of Psychological Events

**DOI:** 10.1371/journal.pone.0042495

**Published:** 2012-08-20

**Authors:** Paul Muentener, Daniel Friel, Laura Schulz

**Affiliations:** Department of Brain and Cognitive Sciences, Massachusetts Institute of Technology, Cambridge, Massachusetts, United States of America; Ecole Normale Supérieure, France

## Abstract

Adults recognize that if event A predicts event B, intervening on A might generate B. Research suggests that young children have difficulty making this inference unless the events are initiated by goal-directed actions [1]. The current study tested the domain-generality and development of this phenomenon. Replicating previous work, when the events involved a physical outcome, toddlers (mean: 24 months) failed to generalize the outcome of spontaneously occurring predictive events to their own interventions; toddlers did generalize from prediction to intervention when the events involved a psychological outcome. We discuss these findings as they bear on the development of causal concepts.

## Introduction

Causal representations are central to human cognition. They support prediction, explanation, and intervention and underlie folk theories across domains [2]–[4]. Moreover, causal representations crosscut conceptual boundaries. Adults are equally adept at reasoning about causal events initiated by an intentional, goal-directed action (e.g., a baseball player hitting a ball), an inanimate object (e.g., a tree falling on a car), or an unobserved entity (e.g., a virus causing a disease). Critically however, developmental studies of causal reasoning have tended to focus only on the first of these contexts: children's inferences in the context of an agent's goal-directed actions. Although considerable research suggests the sophistication of children's causal reasoning even early in development [5]–[15], children are almost uniformly asked to reason about events initiated by dispositional agents (e.g., people or puppets). Investigations of Michottian causality [16] are an important exception to this claim. However, Michottian causality is arguably a modular process, divorced from causal knowledge more broadly [17]–[20]. Thus although causal reasoning in early childhood often appears to be an adult-like, domain-general process, early causal reasoning abilities may, in fact, be predicated upon representations of goal-directed actions.

Consistent with this hypothesis, research has shown that infants' causal reasoning is closely related to their reasoning about intentional agents and their goal-directed actions. Leslie, for instance, showed that 6-month-old infants are sensitive to contact causality between entities in a causal event when the event is agent-initiated, but are insensitive to contact causality when the event is object-initiated [21]. Additionally, infants have a default expectation that agents, not objects, initiate caused motion. When infants see an object emerge from behind a barrier already in motion, infants expect a human hand, and not an object, to be behind the barrier [22], [23]. Even outside of the domain of caused motion, infants' causal inferences appear to be limited to events initiated by goal-directed, intentional agents. When presented with an occluded event in which a box breaks apart or plays music, 8.5-month-old infants expect a relationship consistent with contact causality when the event is initiated by a human hand but not when the event is initiated by an object [24].

The influence of representations of agency on causal reasoning continues beyond early infancy [1], [25]. For example, Bonawitz and colleagues [1] recently showed that even toddlers' causal reasoning is limited outside the context of intentional, goal-directed action. Toddlers were shown several trials of predictive relations in which a block spontaneously moved towards and contacted a base, at which point a toy airplane connected to the base immediately began to spin. For adults, evidence that event A predicts event B suggests the possibility that intervening on A might generate B (i.e., intervening on A to see if B occurs is a good way to learn whether the relationship is genuinely causal). However, although both four-year-olds and toddlers readily learned the predictive relationship, only four-year-olds anticipated the outcome following their intervention (i.e., looked towards the toy after placing the block in contact with the base). Toddlers succeeded only in restricted contexts, in particular when the events were initiated by dispositional agents.

These findings raise questions concerning the nature of the relationship between representations of agency and causality early in development. One possibility is that although toddlers can learn predictive relationships among events [14], [26], and can also learn the relationship between their own and others' interventions and outcomes [27], [28], they do not bind these two kinds of reasoning – reasoning about predictive relationships and reasoning about interventions – into a single, adult-like concept “cause”. Indeed, many researchers have proposed that adult humans may be unique in integrating the kind of predictive reasoning involved in classical conditioning with the ability to anticipate the outcome of interventions characterized by operant learning [6], [29], [30]. Although non-human animals can make different predictions under observation and intervention [31], there is no evidence that animals spontaneously design novel interventions after learning predictive relations. Arguably, this ability develops relatively late, even in human ontogeny. Thus, infants and toddlers' earliest conception of causation may be limited to events involving intentional agents and their goal-directed actions [1], [24].

Alternatively, toddlers might have an adult-like concept of causation but fail to understand some physical mechanisms of as means of causal transmission. Research suggests, for instance, that infants expect causation on contact [24], [32]–[34] and that this expectation persists through early childhood [8]. The conditions under which toddlers fail to form causal representations may violate these early expectations of contact causality. For example, in Bonawitz and colleagues' study [1], the block contacted a base, which was connected to the toy by a bright orange wire. From an adult perspective, a block contacting a base and activating an airplane connected to the base by a wire does not violate contact causality. However, it is possible that toddlers failed to understand the wire as a means of causal contact: the lack of any apparent transformation or visible transmission of force or energy within the wire itself might impede the children's ability to recognize the event as an instantiation of contact causality. Indeed, toddlers were more likely to succeed when the airplane was attached directly to the base and no wire was involved.

This suggests the possibility that toddlers may be more likely to use predictive relations as a basis for establishing a causal representation of an event in domains involving less restrictive transmission relations. In particular, toddlers might more successfully integrate prediction and intervention for psychological causal events, which can occur either through direct contact or (and even more typically) at a distance. By circumventing constraints on toddlers' expectations about mechanisms of causal transmission, toddlers might have no difficulty with the basic task of expecting a predictive relation to be representative of a causal relation.

Consistent with this possibility, previous research suggests that young infants represent many aspects of psychological causal relations. Schlottmann and colleagues, for example, have shown that infants as young as 6 months of age seem to perceive causality in simple social outcomes such as one object chasing another object and causing it to flee [35]–[38]. However, infants' success at representing causal relations in looking-time paradigms does not establish whether the representations underlying such success are causal in the adult-like sense. Although infants may be able to visually discriminate causal from non-causal psychological events, they may not be able to form expectations for the outcomes of their interventions on a psychological event. The current study thus extends previous work on reasoning about psychological causality by investigating whether young children use representations of predictive psychological relations to form expectations about the outcomes of the interventions.

In the current study, we replicate Bonawitz and colleagues' study [1] and compare toddlers' causal reasoning about physical outcomes with their reasoning about psychological outcomes. We present toddlers with predictive events in which a block moves spontaneously towards a base, which is connected to a toy. In the Physical outcome condition, the toy is an airplane that immediately begins to spin; in the Psychological outcome condition, the toy is a puppet who immediately begins to laugh. If toddlers lack a domain-general concept of causation and only integrate prediction and action when events are initiated by agents, they should fail to represent the predictive event as a causal event in both conditions because the block always begins to move spontaneously; agents are never involved in initiating the events. By contrast, if toddlers have a domain-general understanding of causation but simply fail to understand some mechanisms of physical transmission, they should fail in the physical condition but succeed in the psychological condition.

## Procedure, Results, and Discussion

**Figure 1:**
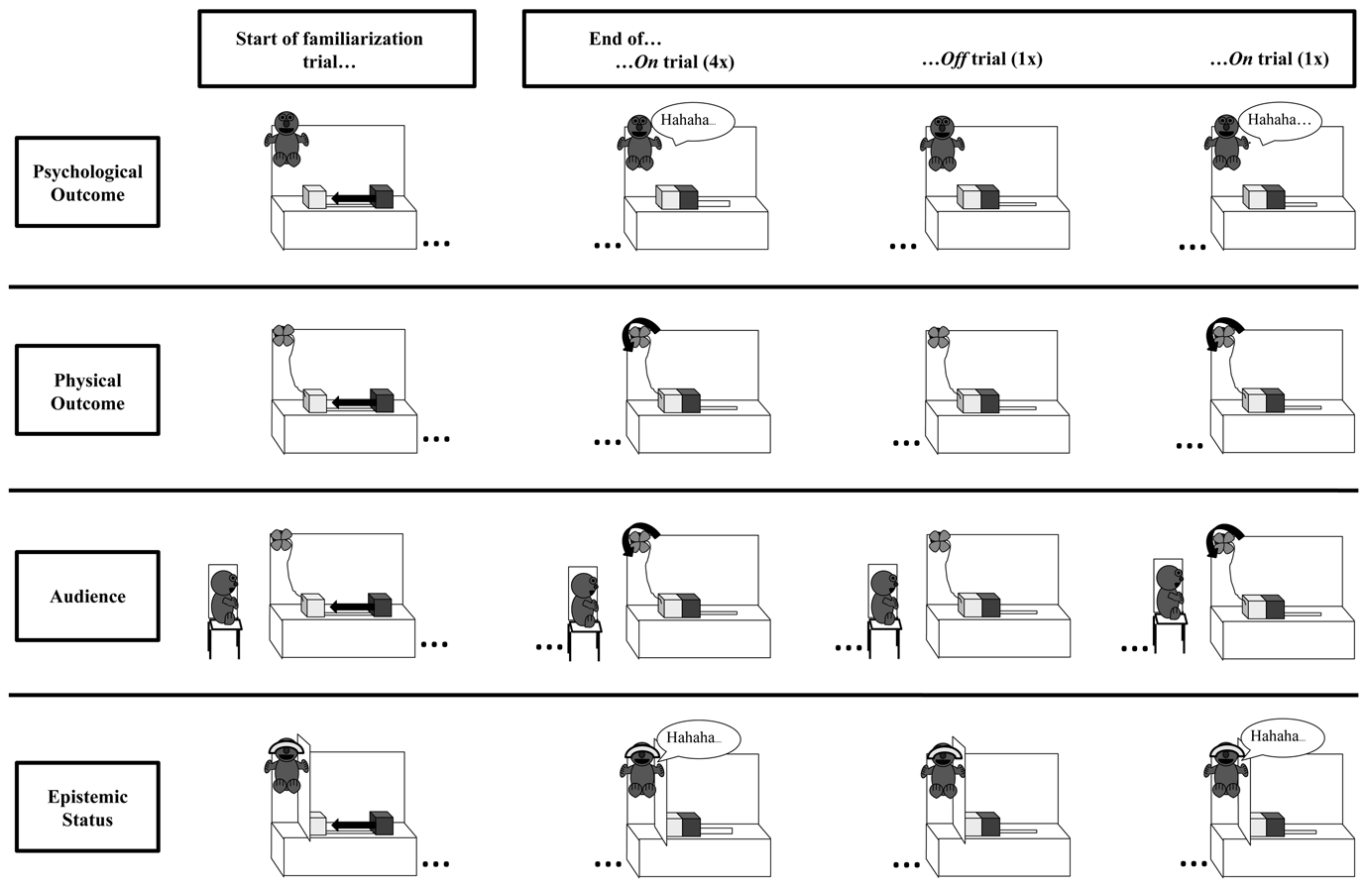
Experimental procedures for the Psychological outcome, Physical outcome, Audience, and Epistemic Status conditions. Toddlers viewed four On familiarization trials, an Off familiarization trial, and a final On familiarization trial. If toddlers failed to look towards the effect in the Off familiarization trial, then they viewed two additional trials (On, Off) before the final On trial. In the Audience condition, the puppet was audience member to the physical outcome event, laughing prior to the start of each trial. In the Epistemic Status condition, there was a wall between the puppet and the blocks, and a blindfold was placed over the puppet's eyes at the start of the experiment.

All toddlers viewed a three-part predictive event in which (1) a block spontaneously began to slide across a stage from rest towards a base block and (2) contacted the base block, after which (3) an effect occurred (see Figure 1). In the Physical outcome condition, a small airplane, which was connected to the base by a wire, began to spin. In the Psychological outcome condition, a puppet began to laugh. Following familiarization with the predictive event, toddlers were given the block and asked to make the effect occur.

The first set of analyses assessed whether toddlers had learned the predictive relationship between the block's motion and the outcome. We assessed toddlers' learning of the predictive event by coding whether toddlers looked up towards the toy in the first 3 seconds following an Off trial during the familiarization phase. During the Off trial, the block contacted the base, but the effect did not occur. If in the experimenter's online judgment (see coding below) toddlers did not look up towards the toy within 3 seconds of the block contacting the base, we presented an additional On trial in which the effect occurred, followed by an additional Off trial. Following each toddler's final Off trial, they viewed one final On trial, to show that the effect had not spontaneously stopped occurring.

All results used for analyses were coded from videotape by two coders blind to conditions. (See Methods.) In all cases where the experimenter introduced a second Off trial the blind coders' judgment agreed with the experimenter's online judgment. Table 1 displays the results for all the analyses described below. Overall, the majority of toddlers in both conditions looked up towards the toy within 3 seconds of the block contacting the base, even though the toy was not activating. We thus concluded that they learned the predictive relationship: in the Physical outcome condition, 18 of the 22 toddlers (81.82%) looked up towards the toy airplane after the block contacted the base; in the Psychological outcome condition, 15 of the 16 toddlers (93.75%) looked up towards the puppet after the block contacted the base. There was no significant difference between the conditions (Fisher's exact test, *p* = n.s.). Of those toddlers that learned the predictive relationship, no toddlers in the Psychological outcome condition and 3 toddlers in the Physical outcome condition needed an additional Off trial (Fisher's exact test, *p* = n.s.). Although there were no significant differences between conditions, children were only included in further analyses if they learned the predictive relationship. This ensured that any differences that emerged during subsequent phases were not due to differential initial encoding of the predictive relationship.

**Table 1:** Results.

		Psychological Outcome	Physical Outcome	Audience	Epistemic Status
Total # of toddlers who participated in each condition		16	22	22	18
**Did toddlers learn the predictive relationship?**					
Learned predictive relationship…		15/16 (93.75%)	18/22 (81.82%)	17/22 (77.27%)	17/18 (94.44%)
	*…during 1st Off trial*	15	15	15	17
	*…during 2nd Off trial*	0	3	2	0
**Of those toddlers who learned the predictive relationship, did they intervene in the event?**					
Intervened…					
	*…spontaneously*	11/15 (73.33%)	7/18 (38.89%)	10/17 (58.82%)	9/17 (52.94%)
	*…after prompt from experimenter*	4/15 (26.67%)	8/18 (44.44%)	5/17 (29.41%)	6/17 (35.29%)
**Of those toddlers who both learned the predictive relationship and intervened on the event, did they look to the outcome?**					
Looked towards outcome		**15/15 (100.00%)** **	**5/15 (33.33%)**	**6/15 (40.00%)**	**5/15 (33.33%)**

** Fisher's Exact, *p*<.01.

The next set of analyses explored toddlers' ability to perform the target action following the predictive events. Toddlers acted spontaneously on the block in both conditions, although there was a trend for more toddlers to act on the block spontaneously in the Psychological outcome condition. Seven of the 18 toddlers (38.89%) in the Physical outcome condition spontaneously placed the block in contact with the base during the test phase. By contrast, 11 of the 15 toddlers (73.33%) in the Psychological outcome condition spontaneously performed the action (Fisher's exact test, *p* = .08). If toddlers failed to act spontaneously, the experimenter prompted them by moving the block part way towards the base. All of the remaining 4 toddlers in the Psychological outcome condition and 8 of the 11 remaining toddlers in the Physical outcome condition completed the action. Three toddlers in the Physical outcome condition failed to perform the action either spontaneously or following a prompt and were thus removed from further analysis.

Although toddlers were marginally more likely to act on the block in the Psychological condition than in the Physical condition, the action by itself is hard to interpret. Toddlers may have been slightly more likely to act spontaneously in the Psychological condition than the Physical condition because they were more likely to treat the events as causal in the Psychological than Physical condition. Alternatively, toddlers might have understood the causal relationship equivalently in both conditions (either failing to infer that *either* relationship was causal or inferring that *both* were) and their tendency to act on the block might reflect only different levels of overall motivation and engagement.

If toddlers truly represent the psychological, but not the physical, outcome events causally, then toddlers should expect their action on the block to bring about the effect only in the Psychological condition. As a result, our primary measure of interest was whether toddlers appeared to expect their own action to generate the outcome. For the 15 toddlers in each condition who both learned the predictive relationship and acted (either spontaneously or after prompting) on the block, we coded whether they looked up towards to the toy within a 3 second window after they placed the block in contact with the base; the toy remained in the OFF position after the toddlers' interventions in both conditions. In the Physical outcome condition, only 5 of the 15 toddlers (33.33%) looked to the toy after intervening. By contrast, in the Psychological outcome condition, all of the toddlers did so (15/15 toddlers; 100.00%; Fisher's exact test, *p*<.0005).

These results suggest that the nature of the outcome (psychological vs. physical) influenced toddlers' tendency to generalize the outcome from the spontaneously occurring event to the consequence of their own intervention. This is consistent with previous research suggesting that toddlers have difficulty representing non-agentive physical predictive relationships as potential causal relationships [1], [25]. Although toddlers learned the predictive relationship between the block and the airplane's motion and acted on the block, toddlers did not look towards the airplane following their action. We infer from this measure that toddlers did not expect their action to cause the event to occur. By contrast, toddlers did represent the predictive relation as a potential causal relationship for the psychological outcome: they looked to see whether their intervention generated the outcome.

Note that we always verified that the toddlers had learned the predictive relationship by including a familiarization trial in which the effect did not occur. Thus all the events presented occurred probabilistically. One possibility is that toddlers are more willing to accept probabilistic relationships for psychological events than physical events. If so, toddlers might generalize the outcome in the Psychological but not Physical condition because they are more willing to accept probabilistic relationships among agents than among objects. We think this interpretation is possible but unlikely to account for the current results. Critically, Bonawitz and colleagues' study [1] included a deterministic physical condition in which the block's motion always resulted in the airplane's spinning. Children's performance did not improve when the physical condition was deterministic; toddlers were no more likely to look towards the airplane following their intervention in the deterministic condition than in the probabilistic conditions.

However, our results do provide some suggestive evidence that the Psychological condition might have been more motivating and engaging than the Physical condition. Although the children were equally likely to learn the predictive relationship in both conditions, there was a non-significant trend for toddlers to act on the block more often in the Psychological outcome condition than in the Physical outcome condition. To the degree that this trend is meaningful, it might (as discussed) be due to different levels of overall engagement with the task. If so, such differential engagement (rather than differential sensitivity to the causal relationships) might account for the condition differences.

To investigate the possibility that children's interest in the puppet contributed to children's different performance in the Psychological and Physical conditions, we ran an Audience condition, intended to increase the children's engagement with the Physical outcome condition. In the Audience condition we introduced a laughing puppet before each *physical* predictive event. The children were instructed to greet the puppet and the puppet laughed at the start of ever familiarization trial; the puppet's laughing was identical to that in the Psychological condition. If the puppet's laughing simply enhances toddlers' arousal or increases their motivation to participate in the task, then toddlers' performance in this condition should improve. However, if, as we hypothesize, toddlers have difficulty generalizing from prediction to intervention for spontaneously occurring physical events, then they should continue to fail to generalize the outcome associated with the predictive event to their own interventions in this condition.

Again, children were counted as learning a predictive relationship if the independent coders judged that the child had look to the (still and silent) puppet within 3 seconds of the block contacting the base. First, we found that the majority of toddlers learned the predictive relationship between the block and the effect (17 of 22 toddlers (77.27%)), no different from either the Psychological outcome or Physical outcome conditions (Fisher's exact tests, *p* = n.s.). Of those toddlers who learned the predictive relationship, two toddlers needed an additional Off trial; there was no significant difference between this condition and either the Physical outcome or Psychological outcome conditions in the number toddlers needing an additional Off trial (Fisher's exact test, *p* = n.s.). Nonetheless, to ensure that subsequent results were not due to toddlers' failure to learn the initial predictive relationship, toddlers who failed to learn the predictive relationship (n = 5) were removed from subsequent analyses.

Toddlers' tendency to act spontaneously on the block in the Audience condition did not differ from either the Psychological outcome or Physical outcome conditions (Fisher's exact test, *p* = n.s.). Ten of the 17 toddlers (58.82%) in the Audience condition spontaneously intervened by placing the block in contact with the base; five additional toddlers completed the intervention following the experimenter's prompted action. The remaining toddlers who never performed the intervention (n = 2) were removed from subsequent analysis.

As noted however, merely acting on the block does not mean that children expected their action to cause the outcome. Thus our final measure of interest again was whether, having learned the predictive relationship and demonstrating their ability to perform the target intervention, toddlers looked towards the outcome following their intervention. Toddlers were significantly less likely to look towards the outcome in the Audience condition than in the Psychological outcome condition (6 of 15 toddlers (40.00%); Fisher's exact test, *p*<.001). Toddlers' performance in this condition was not significantly different from their performance in the Physical outcome condition (Fisher's exact test, *p* = n.s.).

These results suggest that although toddlers might have found the psychological task (involving a laughing puppet) more interesting or arousing than the physical task (involving the airplane), differential attention, motivation, and arousal are unlikely to account for toddlers' different performance in the Psychological outcome and Physical outcome conditions. The presence of a laughing puppet in the Audience condition did not significantly increase toddlers' tendency to generalize the outcome of their interventions from the outcomes learned predictively.

In a final study, we investigated whether constraints specific to the domain of psychological causality affect toddlers' tendency to treat predictive relations as relations that might support effective interventions. Although psychological events are not subject to a contact constraint, they are subject to the epistemic state of the participants. If the puppet cannot see or hear the block's motion and resulting contact, then he shouldn't laugh in response to the event. To test whether toddlers would (appropriately) fail to represent psychologically implausible events causally, we ran an Epistemic Status condition. In this condition, we blocked the puppet's visual access to the block's motion by blindfolding the puppet and placing a wall between the puppet and the blocks. We pointed out that the puppet could not see or hear the events behind the wall. If toddlers' differential performance in the Psychological outcome and Physical outcome conditions is due to the toddlers' understanding of psychological causality, then they should not represent the predictive relation as a causal event when the events are psychologically unlikely.

We found that the majority of toddlers learned the predictive relationship between the block and the effect (17 of 18 toddlers (94.44%)) and that this performance was no different from either the Psychological outcome or Physical outcome conditions (Fisher's exact tests, *p* = n.s.). Of these 17 toddlers, all demonstrated learning of the predictive relationship on the first Off trial (*p* = n.s., compared to the Physical outcome and Psychological outcome conditions). One additional toddler was removed from subsequent analyses for never having learned the predictive relationship on either Off trial.

Toddlers were as likely to intervene in the Epistemic Status condition as they were in the Psychological outcome and Physical outcome conditions (Fisher's exact test, *p* = n.s.). Nine of the 17 toddlers (52.94%) in the Epistemic Status condition spontaneously intervened by placing the block in contact with the base, and six additional toddlers completed the intervention following the experimenter's prompted action. The one remaining toddler, who never performed the intervention, was removed from subsequent analysis.

As in the previous studies, merely acting on the block is not evidence that the children expected their action to cause the outcome. Of the toddlers who learned the predictive relationship and demonstrated their ability to perform the target action, toddlers were significantly less likely to look towards the outcome following their intervention in the Epistemic Status condition than in the Psychological outcome condition (5 of 15 toddlers (33.33%); Fisher's exact test, *p*<.001); toddlers' performance was not significantly different from the Physical outcome condition (Fisher's exact test, *p* = n.s.).

These results suggest that toddlers' causal representations of the psychological event were subject to constraints specific to the domain of psychological causality: toddlers did not represent the predictive psychological outcome as a causal event when the puppet lacked informational access to the block's movement (i.e., in the Epistemic Status condition). Additionally, these results replicate the Audience condition in suggesting that the mere salient, arousing presence of a laughing puppet cannot account for children's different performance in the Physical and Psychological conditions. Therefore, these results suggest that toddlers can reason about causal events involving psychological outcomes even when the initiating events occur spontaneously.

Previous work [1] left open the possibility that toddlers lacked a domain-general concept of causation that integrated prediction and intervention. The current study provides evidence against that view. Toddlers were able to observe a non-agentive predictive relationship and move from learning the predictive relationship to designing an appropriate intervention and anticipating the outcome. Critically however, they only did so when the outcome was a psychological one. Arguably, this is because children do not have an expectation of, and therefore do not perceive any violation of, constraints on contact causality for psychological events; for physical events, invisible transmission (through a wire) might represent an apparent violation of contact causality. That is, toddlers appear to have access to an integrated concept of causation which bridges the gap between prediction and intervention, but the events to which they apply this concept depend on how causation is instantiated in particular domains.

How then do children reason about physical causal events? One speculative possibility is that infants initially recognize agent-initiated events – events involving their own actions or those other goal-directed agents – as causal events [27], [28], [39], [40]–[42]. With respect to non-agentive events, infants might initially apply the concept of causation only to contact causality resulting in object motion [43], [44]. Even recognizing causal relationships for non-agentive contact events involving object changes of state (rather than object motion) may be a later development [24]. By the second year, toddlers may recognize non-agentive causal relationships as long as there is a continuous, visible transmission of force or energy [45]. Only relatively late in development may children realize that they can engage in causal reasoning for a larger class of events, including non-agentive events that occur without visible transmission of energy or information (e.g., through wires or even through invisible connections).

Future work might investigate this developmental story about how children understand mechanisms of physical transmission. Even if an account like this is correct however, the question remains of why children readily accept the entire range of these transmission events as causal, as long as goal-directed agents initiate the events. Additional research might look at whether children can bootstrap from their understanding of the goal-directed causal events to their understanding of means of transmission in the absence of dispositional agency.

Another outstanding question from this research concerns the extent of toddlers' understanding of psychological outcomes. The current study investigated only one type of psychological outcome (i.e., laughing). However, the class of events that encompass the domain of psychological outcomes is vast. Events can make us laugh, cry, perform an action, or inhibit a response. Moreover, philosophers have long suggested that we may perceive psychological outcomes as “uncaused” insofar as we believe they are generated by the agent's free will; thus adults often draw a distinction between reasons for actions and causes for actions [46]. Whether there is any sense in which infants and toddlers are sensitive to the distinction between reasons and causes (and the developmental trajectory of this distinction) remain areas ripe for future inquiry.

The current results suggest that, although children have an adult-like abstract understanding of the concept of causation that binds prediction and action by two years of age, their ability to recognize particular events as instances of causation may depend on domain-specific constraints. Sensitivity to these constraints may develop in a piecemeal manner, in which viable means of causal transmission are learned event-by-event. This study thus adds to a growing body of research suggesting that in tandem with or even before they have an accurate understanding of the specific causal mechanisms, young children have a rich, abstract understanding of causality [4], [9], [47]. In some contexts, this understanding allows even toddlers to perform novel interventions and accurately predict the outcome of their own actions on the world.

## Materials and Methods

### Ethics Statement

The Massachusetts Institute of Technology Institutional Review Board approved the procedures for all research described in this paper. We obtained written consent from the participants' parents.

### Participants

Seventy-eight toddlers (mean: 24.5 months, range: 18–30 months) were recruited at two children's museums. Toddlers were assigned to one of four conditions: a Psychological outcome condition (n = 16), a Physical outcome condition (n = 22), an Audience condition (n = 22), or an Epistemic Status condition (n = 18). Note that participants were recruited to match in the final sample included for analysis (n = 15/condition); different n's in condition assignment reflect non-significant differences in the number of participants failing to meet the inclusion criteria described below. An additional 10 toddlers were recruited but not included in the final sample due to: inability to complete the session (n = 5; Psychological: 1, Physical: 2, Epistemic Status: 2), parental interference (n = 3; Psychological: 1, Audience: 1, Epistemic Status: 1), or experimenter error (n = 2; Physical: 1, Audience: 1). There were no age differences between the conditions (*p* = n.s.).

### Materials

All events occurred on a white stage (30 in.×12 in.) that blocked a confederate from view (See Figure 1.) A blue block (the “base”, 1 ¾ in.×2 in.×3 ½ in.) and a green block (1 ½ in.×1 ½ in.×2 ½ in.) were on opposite ends of the stage. The green block was attached to a stick extending through the floor of the stage, allowing the hidden confederate to surreptitiously move the block across the stage to the base. In the Physical outcome condition, a toy airplane, attached to the base by a wire, was located on the back stage wall. In the Psychological outcome condition, a puppet with eyes was seated on a perch on the back stage wall. The confederate controlled the actions of the airplane and puppet. In the Epistemic Status condition, there was also a blindfold on the puppet so that the puppet's eyes and ears were covered. In addition, a wall (20 in.) was placed between the puppet and the base so that the puppet had no visual access to the block or base.

### Procedure

#### Psychological outcome condition

The experiment had two phases: a familiarization phase and a test phase. There were two types of familiarization trials: On trials and Off trials (see Figure 1). Each toddler first viewed four On trials, in which the block began at the far right of the stage. The experimenter drew the toddler's attention to the stage saying, “Watch my show.” The block then moved spontaneously towards and contacted the base. As soon as the block contacted the base, the puppet laughed and wiggled for three seconds. At the end of the On trial, the stage was covered by an occluder, and the scene was reset. Following the On trials, the toddlers viewed one Off trial. The Off trials were identical to the On trials except that the puppet did not laugh. The experimenter ended the Off trial after the toddler looked towards the puppet or after three seconds – whichever came first. If toddlers did not look towards the effect during the Off trial, the experimenter repeated another On trial, followed by another Off trial. All toddlers then viewed one final On trial. Thus, if toddlers looked towards the outcome on the first Off trial, they saw a total of six trials; if they required a repetition, they saw a total of eight trials.

At the start of the test phase, the experimenter handed the block to the toddler and asked the child to make the effect occur. If the child did not spontaneously place the block in contact with the base, the experimenter prompted the toddler to place the block in contact with the base. The prompt involved the experimenter pushing the block across the stage towards, but stopping just short of, the base block. The experimenter then handed the block back to the toddler and encouraged them to make the effect occur.

#### Physical outcome condition

The Physical outcome condition was identical in structure (4 On trials, 1 Off trial, 1 On trial, 1 possible Off trial, 1 possible On trial) to the Psychological outcome condition except that instead of a puppet laughing after the block's movement, a toy airplane spun for three seconds at the top left corner of the stage (see Figure 1).

#### Audience condition

The Audience condition mirrored the Physical outcome condition. Additionally, the puppet used in the Psychological outcome condition was placed on the experimenter's lap and acted as an audience member with the toddler to the familiarization events. At the start of each familiarization trial (both On trials and Off trials), the experimenter drew the child's attention to the puppet, who was seated next to the stage, on the experimenter's lap. The experimenter asked the child to say hello to the puppet, and the puppet then laughed and wiggled for three seconds (exactly as in the Psychological outcome condition). After the puppet laughed, the experimenter told the child that the puppet was going to watch the show with them and then turned the puppet to face the stage. The puppet laughed and turned towards the stage in an identical manner prior to the start of every familiarization trial. We had the puppet laugh before the trials rather than after so that the puppet's laughter could not be construed as an effect; we had the puppet laugh on the experimenter's lap rather than on the stage so the puppet could not be construed as a dispositional agent initiating the events. The trials then proceeded as On trials or Off trials, which mirrored the Physical outcome condition. During the test phase, the puppet did not laugh. The experimenter gave the block to the child and asked them to make the effect occur.

#### Epistemic Status condition

The Epistemic Status condition was identical to the Psychological outcome condition, except as follows. Prior to the start of the familiarization phase, the experimenter placed a blindfold over the puppet's eyes. The experimenter then told the toddler that the puppet could not see or hear what was happening during the study (“I'm going to cover his eyes and his ears so that he can't see or hear what is happening during the show.”). Additionally, a wall was placed between the puppet and the blocks (prior to the child entering the room), such that the puppet had no visual access to the predicting event. The remainder of the familiarization phase was identical to that of the Psychological outcome condition. The block spontaneously moved towards and contacted the base, after which the puppet laughed for 3 s.

#### Coding

Following data collection, two raters, blind to experimental condition, independently scored toddlers' behaviors. 75% of responses were double-coded; inter-rater agreement was high (93.33%, kappa = .866).
